# In Situ Casting Integrated with FDM 3D Printing: Curing Behavior, Process Constraints, and Mechanical Demonstration

**DOI:** 10.3390/polym18081003

**Published:** 2026-04-21

**Authors:** Supatpromrungsee Saetia, Pimolkan Piankitrungreang, Ratchatin Chancharoen

**Affiliations:** 1 Department of Mechanical Engineering, Faculty of Engineering, Chulalongkorn University, Bangkok 10330, Thailand; 6772104321@student.chula.ac.th (S.S.); 6770266021@student.chula.ac.th (P.P.); 2 Human-Robot Collaboration and Systems Integration Research Unit, Chulalongkorn University, Bangkok 10330, Thailand

**Keywords:** in situ casting, polymer 3D printing, dispensing-based extrusion, hybrid additive manufacturing

## Abstract

Dispensing-based in situ casting offers a practical route for introducing dense or mechanically distinct polymer regions into fused deposition modeling (FDM) parts during fabrication. This study investigates the curing-dependent process constraints governing stable integration of in situ casting within an FDM workflow. In the proposed process, FDM is used to fabricate thermoplastic confinement geometries, after which liquid polymer is dispensed into selected cavities and cured before printing resumes. Two representative curing systems were examined: a UV-curable photopolymer and a two-component epoxy resin. The experimental program included UV curing characterization under perpendicular 405 nm exposure, infrared thermal imaging of curing-induced heat generation and dissipation, confined curing of epoxy resin, layer-wise integration within an FDM-printed cavity, and a representative mechanical linkage demonstration. The results show that UV-based in situ casting is constrained by the coupled effects of curing depth, peak temperature, and visible deformation, making staged curing with intermediate thermal relaxation necessary for stable operation. In contrast, the epoxy system enabled bulk cavity filling with lower peak temperature, but required substantially longer curing time, introducing a different process limitation. A layer-wise UV curing strategy enabled successful stacking of four cast layers within an FDM-printed confinement without visible leakage or shell collapse. Mechanical testing of hybrid linkage specimens further showed that localized casting can modify structural stiffness through selective reinforcement. These findings demonstrate that dispensing-based in situ casting can be integrated into FDM when thermal, temporal, and filling constraints are explicitly managed, and they provide practical process guidance for hybrid polymer fabrication involving confined casting during printing.

## 1. Introduction

Fused deposition modeling (FDM) is one of the most widely used polymer additive manufacturing processes because of its low tooling requirement, geometric flexibility, and compatibility with digital design workflows [1,2,3]. Despite these advantages, the layer-wise deposition of thermoplastic filaments inherently introduces limitations such as stair-stepping, anisotropic mechanical behavior, and internal void formation [4,5,6,7]. These limitations become more pronounced when applications require dense local reinforcement, smooth internal features, or region-specific material functionality [2,8].

To overcome these limitations, hybrid fabrication strategies that combine FDM with secondary deposition, cavity filling, or embedded materials have attracted growing attention [9,10,11,12,13,14,15]. Among these, dispensing liquid polymers into printed cavities provides a practical route for introducing dense or mechanically distinct regions into FDM parts. When such filling is performed during fabrication rather than as a post-process, the workflow follows a print–pause–dispense–cure–resume sequence, in which the printed thermoplastic structure acts as both confinement and support for continued build progression.

A broader comparison of additive, subtractive, and formative manufacturing routes further illustrates this trade-off, as summarized in Table 1. Extrusion-based FDM enables direct digital fabrication with broad thermoplastic accessibility and minimal pre-process burden [16], but typically provides lower surface quality and less uniform mechanical performance than casting or machining. Stereolithography (SLA) improves surface finish through layer-wise photopolymerization [17,18,19], but remains constrained by resin chemistry and curing requirements. Subtractive processes such as CNC machining offer high dimensional precision and excellent surface finish [8], but are fundamentally material-removal approaches and are less suited to directly producing enclosed internal polymer features. Formative processes such as casting can generate dense regions with comparatively smooth surfaces and favorable local mechanical properties, but they usually depend on dedicated molds and are less naturally integrated into layer-wise digital fabrication workflows. These contrasts motivate hybrid approaches that combine the geometric flexibility of FDM with the local surface and property advantages of confined casting.

The main challenge is that stable in situ casting depends not only on cavity geometry, but also on curing behavior, thermal response, and process timing. For UV-curable systems, cured depth is limited by optical attenuation, while rapid polymerization may generate localized heating and deformation [20,21,22]. For two-component systems, optical limitation is absent, but curing is slower and may still introduce heat generation and interruption of the printing sequence [17,23,24]. In both cases, successful integration requires that the dispensed material be cured within the printed cavity without leakage, shell distortion, or unacceptable delay to the FDM process.

Although related approaches have been reported in print-and-fill, embedding, and hybrid dispensing workflows [9,10,11,12,13,14,15,25], experimentally grounded guidance remains limited regarding how curing mechanisms, thermal evolution, dispensed volume, and process timing interact during in situ casting within FDM. As a result, process implementation is often empirical rather than systematically designed.

This study experimentally investigates dispensing-based in situ casting integrated within an FDM workflow, with emphasis on the curing-dependent constraints governing stable operation. Two representative polymer systems were examined: a UV-curable photopolymer and a two-component epoxy resin. The objectives were to determine: (i) the curing depth, thermal, and deformation constraints of UV-based in situ casting, (ii) the thermal and temporal differences between UV and epoxy systems, (iii) the conditions required for stable layer-wise integration, and (iv) the structural relevance of the approach in a representative linkage component.

The aim of this work is not to replace FDM, SLA, CNC, or conventional casting as standalone processes [8,11,18,26,27], but to identify how confined casting can be integrated into an FDM workflow to exploit complementary process advantages within a single build. The contributions are threefold. First, the study identifies curing-dependent process constraints relevant to in situ casting during FDM. Second, it demonstrates a practical layer-wise integration strategy for UV-curable systems. Third, it shows, through a representative linkage case, that confined casting can modify local structural response in a hybrid polymer part.

## 2. Process Description and Study Scope

To examine how dispensing-based in situ casting can be integrated into fused deposition modeling (FDM), it is first necessary to define the process sequence, the governing practical constraints, and the boundaries of the present analysis. In the hybrid workflow studied here, a thermoplastic structure is printed by FDM to create the required confinement geometry [1,7,9,22], after which a liquid polymer is dispensed into selected cavities and cured before deposition resumes. Because successful integration depends not only on geometry but also on curing behavior, thermal effects, and material delivery, this section outlines the workflow considered in the study, identifies the main process constraints that define its feasible operating window, and clarifies the experimental scope and limitations of the present work.

### 2.1. In Situ Casting Within an FDM Workflow

In the process considered here, FDM is first used to fabricate the thermoplastic shell or cavity geometry [15,22]. A liquid polymer is then dispensed into the printed confinement and cured before printing resumes. The workflow therefore follows a print–pause–dispense–cure–resume sequence. The printed structure acts as the cavity boundary, mechanical support during curing, and substrate for subsequent FDM deposition [25].

Unlike conventional FDM, where shape is generated directly by filament placement, the dispensed polymer fills a predefined cavity and is shaped mainly by confinement. The function of dispensing is therefore to deliver a controlled volume at the appropriate stage of fabrication, while curing stabilizes the filled region sufficiently for the build to continue.

### 2.2. Process Constraints

Four practical constraints were considered. First, curing depth is critical for UV-curable resins because only a limited thickness can be reliably solidified under a given exposure. Second, thermal response during curing can affect the surrounding thermoplastic shell and delay print continuation [28,29]. Third, deformation during curing may produce warping, edge lifting, or leakage [10,11,30]. Fourth, volumetric filling control is required because underfilling leaves voids, whereas overfilling may cause overflow or shell distortion. These constraints define the practical operating window for in situ casting during FDM.

### 2.3. Scope of the Present Study

This work is limited to two representative curing mechanisms, namely a UV-curable photopolymer and a two-component epoxy resin [17,22,24]. Simple cavity geometries were used intentionally in the fundamental experiments to isolate curing depth, thermal evolution, and visible deformation. The study emphasizes experimentally observable process behavior rather than full interfacial fracture, rheological, or kinetic modeling. The dispensing platform was used as a practical experimental tool and was not benchmarked against all alternative metering architectures. The mechanical case study is included as a representative demonstration rather than a full qualification campaign.

## 3. Materials and Methods

This section describes the materials, fabrication platform, specimen design, and experimental procedures used to evaluate dispensing-based in situ casting within an FDM workflow. The methods were designed to isolate the key process variables identified in Section 2, namely curing depth, thermal response, visible deformation, and volumetric filling behavior. Two representative polymer systems were examined: a UV-curable photopolymer and a two-component epoxy resin. The experimental program included curing characterization, infrared thermal monitoring, layer-wise integration, and a representative mechanical demonstration.

### 3.1. Materials

ABS and PETG were used as the FDM thermoplastics for shells and confinement structures. A commercial UV-curable photopolymer resin (Anycubic Standard Resin, Green (Anycubic, Shenzhen, China)) [17,22,31,32] was used as the representative photochemical system. A two-part epoxy resin (RA Clear Coat Epoxy Resin 200 (RUNGART, Bangkok, Thailand)) [24,33,34,35] was used as the representative chemically cured system. For the mechanical demonstration, brass bush inserts and selected polymer reinforcement materials were incorporated into printed linkage cavities.

### 3.2. Hybrid Fabrication Platform

**Figure 1:**
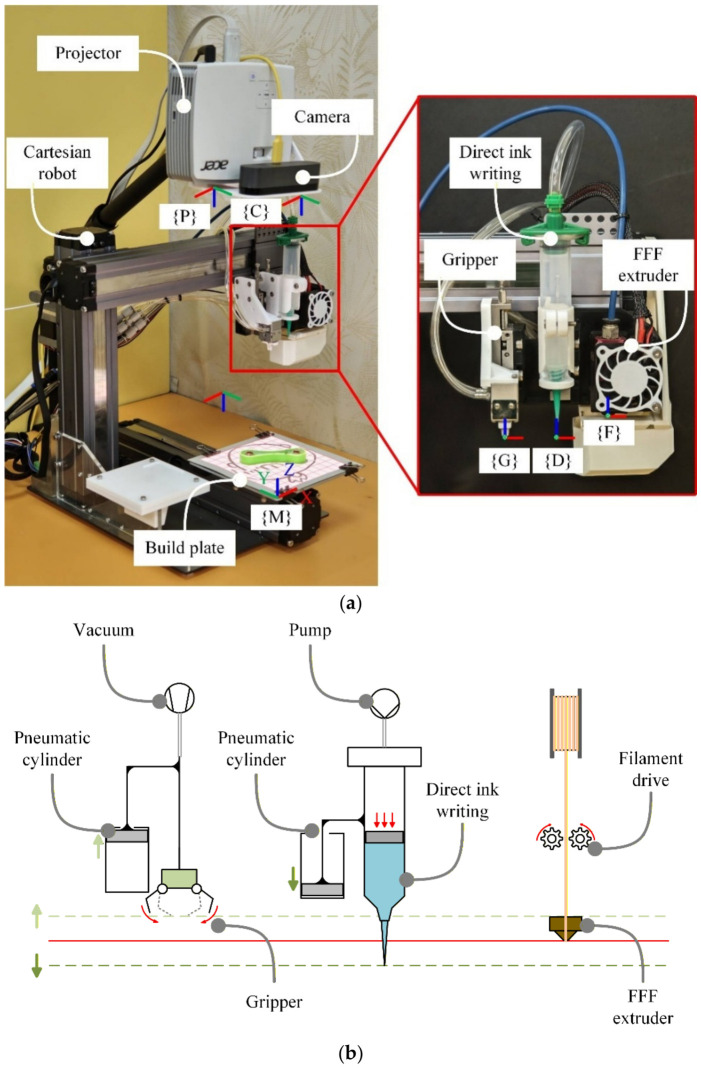
Multi-process fabrication platform for dispensing-based in situ casting: (**a**) integrated system prototype; (**b**) modular multi-toolhead architecture enabling coordinated FDM, dispensing, and auxiliary operations.

Experiments were performed on a multi-process fabrication platform developed for dispensing-based in situ casting within an FDM workflow (Figure 1). Figure 1a shows the integrated system prototype, while Figure 1b illustrates the modular multi-toolhead architecture enabling coordinated FDM deposition, liquid-polymer dispensing, and auxiliary operations within a single build environment.

#### 3.2.1. Dispensing Subsystem

**Figure 2:**
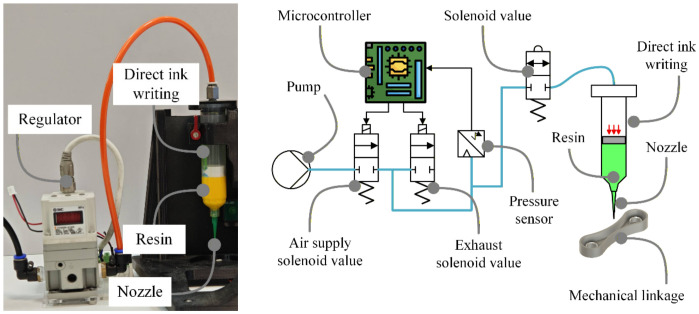
Dispensing-based extrusion subsystem with dual-mode electro-pneumatic actuation and volumetric flow or pressure control.

The dispensing subsystem is shown in Figure 2. The system employed syringe-based material delivery with electro-pneumatic closed-loop pressure regulation and a fixed-geometry nozzle [15,23,36,37]. This configuration enabled repeatable dispensing of both the UV-curable resin and the two-component epoxy into printed confinement geometries under conditions relevant to in situ casting.

#### 3.2.2. UV Curing Subsystem

**Figure 3:**
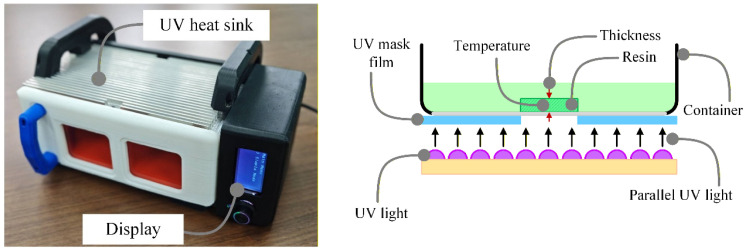
Programmable UV curing oven with digitally controlled exposure intensity and timing for in situ casting experiments.

UV curing was performed using a programmable 405 nm illumination unit [17,31,38,39], shown in Figure 3. The system provided digitally controlled exposure intensity and timing, enabling both single-step exposure and staged curing schedules for the layer-wise integration experiments.

#### 3.2.3. Thermal Imaging Setup

**Figure 4:**
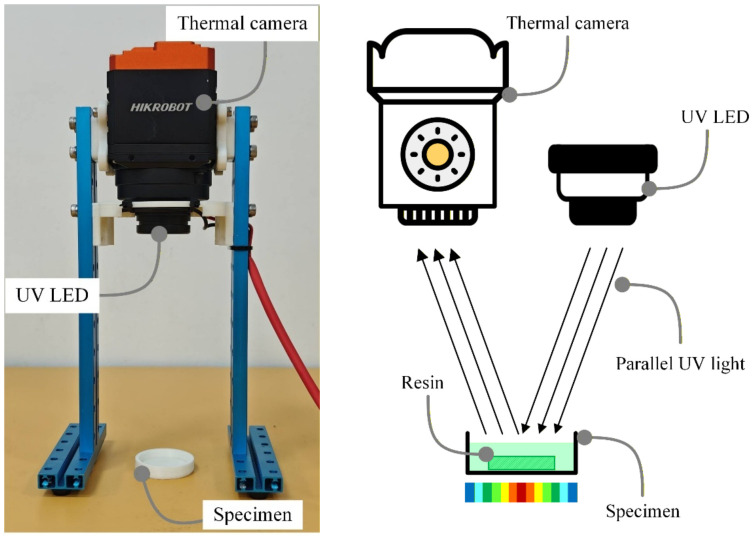
Experimental test rig for UV illumination and infrared thermal imaging used to characterize curing behavior and temperature evolution.

Thermal response during curing was characterized using the experimental rig shown in Figure 4. The setup combined UV illumination with long-wave infrared thermal imaging to capture peak temperature, spatial temperature distribution, and post-exposure cooling behavior during curing. Temperature was measured using a HIKROBOT MV-CI003-GL-N15 thermal camera from Hangzhou Hikrobot Co., Ltd., Hangzhou, China, (resolution: 640 × 512 pixels; operating temperature range: −30 °C to 60 °C; measurement accuracy: ±2 °C or ±2% of reading). Cured thickness was measured from the solidified specimens after exposure under each test condition.

### 3.3. Specimen Design

Simple FDM-printed confinement geometries were used in the fundamental experiments to isolate curing depth, thermal evolution, and visible deformation. For layer-wise integration, a circular mold with a diameter of 30 mm, a height of 5 mm, and a wall thickness of 2 mm was used. For the mechanical demonstration, a linkage-type specimen with local cavities for reinforcement and bush insertion was selected as a representative structural component.

### 3.4. Experimental Procedures

UV curing characterization was carried out under perpendicular 405 nm illumination while varying exposure duration and relative intensity. Cured thickness, peak temperature, and visible deformation were recorded.

Thermal imaging experiments were performed during UV curing to determine peak temperature, spatial temperature distribution, and cooling behavior after exposure.

For the epoxy system, mixed resin was dispensed into the printed confinement and monitored thermally during curing to determine peak temperature, curing timescale, and qualitative fill uniformity.

After UV exposure under each test condition, the resulting cured specimen thickness was measured using a Vernier caliper, while temperature was recorded during the curing process.

Layer-wise integration experiments were conducted by sequentially filling the circular mold with 1 mL of UV resin per layer, followed by staged curing using short pulses of increasing relative intensity separated by dwell intervals. Additional trials included forced airflow to reduce cooling time. Four layers were stacked in total.

For the mechanical demonstration, linkage specimens were first printed by FDM, then locally reinforced using polymer filling and brass bush inserts. The specimens were tested under uniaxial tensile loading, and force–displacement data were recorded.

## 4. Results

### 4.1. Experiment 1: UV Curing Characterization Under Perpendicular Exposure

This experiment investigates the curing behavior of a UV-curable resin under perpendicular 405 nm illumination, with the objective of quantifying the coupled effects of exposure conditions on cured thickness, thermal response, and warping behavior. Exposure time and relative intensity were varied systematically, and curing outcomes were evaluated in terms of cured layer thickness, peak temperature, and deformation severity. The results are interpreted explicitly with reference to the governing constraints derived in Section 5.

#### 4.1.1. Cured Thickness and Exposure Dependence

Across all tested conditions, cured thickness increased with cumulative UV exposure. Short exposures at low intensity produced thin cured layers, approximately 0.35–0.40 mm at 5–10 s and 10% relative intensity. Increasing either exposure duration or intensity yielded thicker cured regions, reaching approximately 1.1–1.5 mm under aggressive exposure conditions. However, thickness growth exhibited diminishing returns at higher exposure levels, indicating a clear saturation trend.

This behavior is consistent with the optical attenuation model presented in Section 5.1. According to the Beer–Lambert relation (Equation (1)) and the dose-threshold condition for polymerization (Equation (2)), the maximum achievable curing depth is expected to follow a logarithmic dependence on exposure dose (Equation (3)). Consequently, increases in exposure intensity or duration are expected to produce progressively smaller gains in curing depth once attenuation becomes significant. The experimentally observed saturation is therefore consistent with an attenuation-limited curing response within the tested conditions.

**Figure 5:**
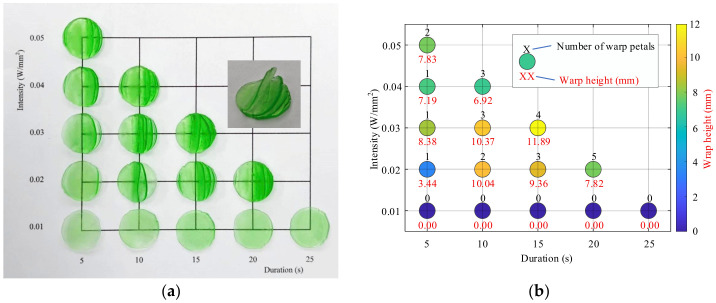

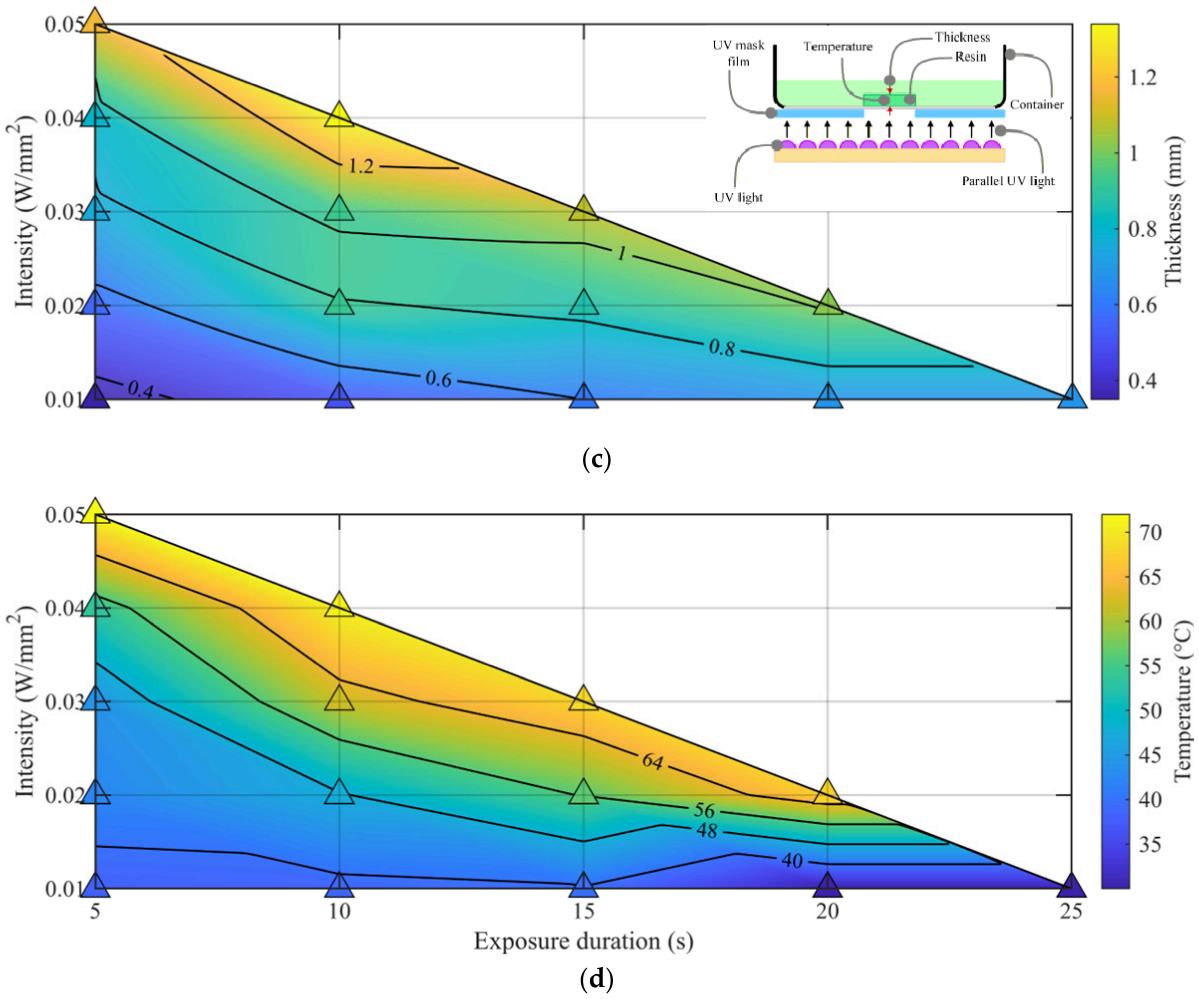
UV curing behavior under perpendicular 405 nm exposure. (**a**) Representative cured specimens obtained under different combinations of relative light intensity and exposure duration. (**b**) Post-cure deformation metrics, reported as warp height and petal-like deformation count for each specimen. (**c**) Contour map summarizing the dependence of cured thickness on relative light intensity and exposure duration. (**d**) Contour map summarizing the corresponding dependence of peak temperature on relative light intensity and exposure duration. Together, these results illustrate the coupled effects of exposure conditions on curing extent, thermal loading, and curing-induced deformation.

The results shown in Figure 5a–c indicate that, within the tested exposure range and geometry, curing thickness does not increase proportionally with exposure time indefinitely. Rather than validating a general theoretical limit, these observations support a practical interpretation that thin, layer-wise curing increments are preferable for maintaining dimensional control.

#### 4.1.2. Thermal Response During UV Curing 

The thermal response during UV curing was evaluated by measuring the peak temperature of the resin specimen under different combinations of UV intensity and exposure duration. The corresponding results are shown in Figure 5b,d. In general, the measured peak temperature increased with exposure severity. At low-intensity conditions, peak temperatures remained below approximately 40–45 °C, even at longer exposure durations, whereas moderate-to-high intensities produced much higher peak temperatures, exceeding approximately 65–70 °C under relatively short exposures.

As shown in Figure 5b,d, peak temperature increased with exposure severity, with UV intensity exerting a stronger influence than exposure duration. This trend is consistent with the transient energy balance described in Section 5.2 (Equation (4)). Low-intensity conditions maintained temperatures below approximately 40–45 °C even for extended exposure times, whereas moderate-to-high intensities produced rapid temperature rises, with peak temperatures exceeding 65–70 °C under relatively short exposures.

Under high-intensity illumination, the curing rate ∂ϕ/∂t increases sharply, amplifying the exothermic heat-generation term relative to conductive dissipation. As a result, peak temperature is governed primarily by instantaneous energy input rather than total exposure duration. By contrast, lower-intensity or temporally segmented exposure permits partial heat dissipation between curing increments, thereby reducing thermal accumulation.

The experimental results are consistent with the interpretation that UV intensity strongly influences peak temperature and therefore defines an important thermal compatibility constraint when integrating in situ casting with thermoplastic FDM layers. The thermal behavior is likewise consistent with an interpretation in which boundary heat transfer plays an important practical role under the tested conditions. However, the underlying heat-transfer mechanisms were not directly measured in the present study and should therefore be interpreted cautiously.

#### 4.1.3. Warping Behavior and Deformation Modes

Visible deformation, as shown in Figure 5a,b was observed under some of the tested curing conditions. In general, specimens associated with thinner cured layers and lower peak temperatures showed little or no obvious warping, whereas more pronounced deformation was more frequently observed under conditions associated with greater cured thickness and higher peak temperature. The observed deformation mainly appeared as edge lifting and petal-like out-of-plane distortion of the cured specimen. In several cases, these deformation features became visually apparent when the cured thickness approached approximately 0.8–1.0 mm.

These observations suggest that deformation tendency increased with curing severity under the tested conditions. A possible interpretation is provided by the shrinkage-related stress framework introduced in Section 5.3, in which polymerization within a constrained geometry may generate internal stress that contributes to visible warping. However, because shrinkage strain, interfacial stress, and delamination were not measured directly in the present study, this explanation should be regarded as qualitative and interpretive rather than as a direct experimental finding.

The observed deformation also did not appear to depend on temperature alone. Some conditions associated with moderate temperature rise still showed visible warping, whereas certain higher-temperature conditions showed less pronounced petal-like distortion. This suggests that the final deformation pattern may reflect the combined influence of multiple factors, including cured thickness, thermal history, shrinkage, and local boundary conditions at the resin–substrate interface. Once deformation begins, the resulting morphology may also be influenced by resin redistribution beneath the cured region during continued exposure.

#### 4.1.4. Implications for In Situ Casting

The results in Section 4.1.1, Section 4.1.2 and Section 4.1.3 indicate that exposure conditions promoting greater cured thickness also tended to produce higher peak temperature and greater visible deformation. In particular, increasing UV intensity and exposure duration increased the achievable curing depth, but also increased the thermal load and the likelihood of instability in the cured region. These combined observations suggest that reliable UV-based in situ casting requires practical coordination of both curing depth and thermal loading, rather than optimization of exposure dose alone.

In the present context, control of curing depth refers to limiting the thickness cured in each step through suitable selection of intensity, exposure duration, and fill thickness, whereas control of peak temperature refers to limiting thermal accumulation through reduced exposure severity, segmented curing, and adequate dwell time for cooling. The results therefore support the use of layer-wise UV curing with intermediate thermal relaxation to maintain process stability and compatibility with subsequent FDM deposition.

### 4.2. Experiment 2: Heat Generation and Dissipation During UV Curing

**Figure 6:**
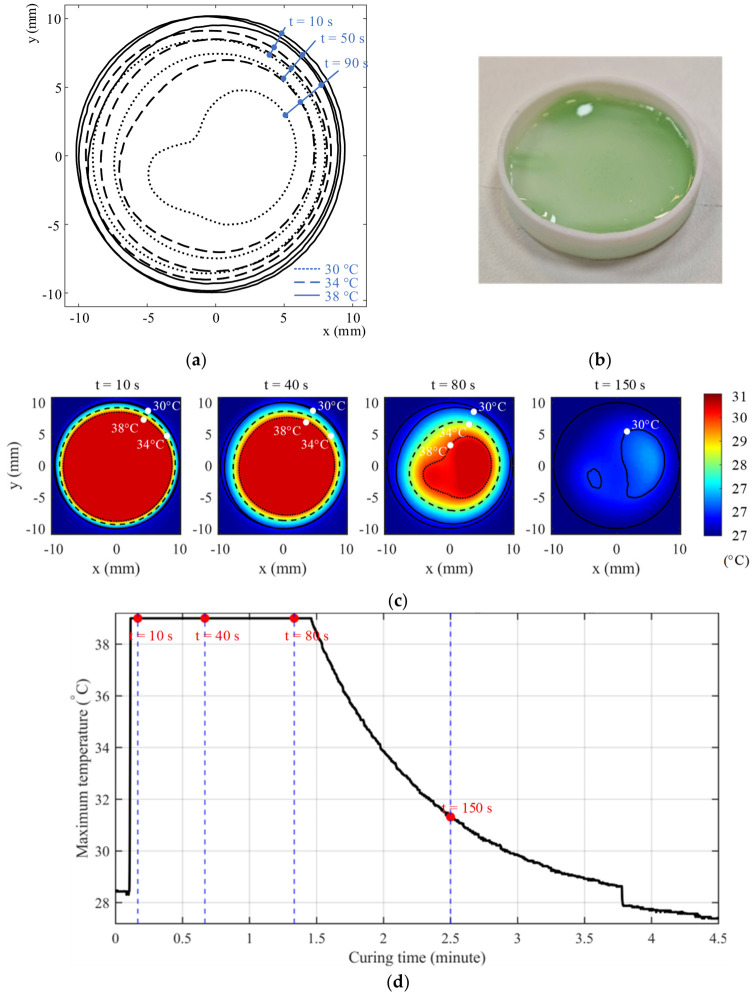
Spatiotemporal thermal response of a UV-curable resin under perpendicular 405 nm illumination. (**a**) Time-resolved temperature distribution during and after exposure, showing the evolution of localized heating within the confined region. (**b**) Photograph of the test specimen used for thermal characterization. (**c**) Temperature evolution as a function of time, capturing both the heating and post-curing cooling stages. (**d**) Maximum temperature recorded during the curing cycle. Together, these results illustrate localized heat generation, radial temperature gradients, and post-exposure thermal dissipation under confinement provided by the FDM structure.

This experiment examines the spatiotemporal evolution of curing-induced heat during UV exposure, with the objective of characterizing heat generation, diffusion, and dissipation within an FDM-fabricated confinement. Infrared thermal imaging was used to quantify temperature distributions and temporal decay following UV curing. The spatiotemporal thermal evolution is shown in Figure 6. The results are interpreted with reference to the transient energy balance and thermal boundary conditions described in Section 5.2. Note that the results in this section were derived from analysis of time-resolved infrared thermal images acquired during and after UV curing. The thermal maps were used to examine peak temperature distribution, radial temperature gradients, and the temporal reduction in the heated region during cooling.

#### 4.2.1. Peak Temperature and Spatial Distribution

Immediately following UV exposure, the cured resin exhibited a temperature rise to approximately 40 °C, with a non-uniform spatial distribution. Thermal images consistently revealed a radial temperature gradient, with the highest temperatures localized near the center of the cured region and progressively lower temperatures toward the periphery.

This spatial distribution is consistent with the energy balance formulation in Equation (4), in which heat generation occurs within the resin while heat dissipation is influenced by conductive transport through the surrounding boundaries. The FDM-fabricated confinement therefore appears to establish non-uniform thermal boundary conditions, leading to preferential heat dissipation through the mold walls.

#### 4.2.2. Temporal Evolution of Heated Area

Analysis of isothermal contours showed that the area exceeding selected temperature thresholds decreased monotonically with time following UV exposure. Higher-temperature regions contracted rapidly, while lower-temperature regions persisted over a broader area.

This behavior reflects the dominance of diffusive heat dissipation once UV exposure ceases. As the exothermic source term diminishes after curing completion, temperature evolution becomes governed primarily by thermal diffusion into the surrounding FDM structure, as captured by the Laplacian term in Equation (4).

#### 4.2.3. Role of FDM Confinement in Thermal Dissipation

The persistent radial gradients observed across all conditions indicate that the printed FDM confinement actively participates in thermal management. Heat flux is directed outward from the curing resin into the surrounding thermoplastic shell, suggesting that confinement geometry simultaneously defines shape and thermal boundary conditions.

This observation supports the practical interpretation that, under the tested conditions, boundary heat transfer through the surrounding FDM confinement plays a dominant role in thermal dissipation.

#### 4.2.4. Implications for Layer-Wise Fabrication

Although curing-induced heat dissipates within tens of seconds under controlled conditions, localized high-temperature regions persist immediately after exposure. Without sufficient dwell time, cumulative heating may occur during successive curing steps.

These results reinforce the thermal compatibility constraint T ≤ T_crit in the combined process window and provide an experimental basis for the dwell-time requirements adopted in the layer-wise curing strategies presented in Section 5.

### 4.3. Experiment 3: Two-Component Resin Curing Behavior

This experiment investigates the curing behavior of a two-component epoxy resin under confined conditions, with the objective of characterizing temperature evolution, curing timescale, and material uniformity. The results are interpreted in contrast to UV-curable systems and evaluated with respect to the thermal and interfacial constraints defined in Section 5.2 and Section 5.3.

#### 4.3.1. Thermal Response During Two-Component Resin Curing

Curing of the two-component epoxy resin was accompanied by a modest temperature rise, with peak temperatures remaining substantially lower than those observed during UV curing. The maximum recorded temperature occurred gradually and was followed by slow thermal relaxation over several hundred seconds.

This behavior is consistent with the energy balance in Equation (4), with the key distinction that the heat-generation term ΔH(∂ϕ/∂t) is distributed over an extended timescale due to chemically governed reaction kinetics. As a result, peak thermal loading on the surrounding FDM structure remains low.

#### 4.3.2. Curing Timescale and Process Implications

**Figure 7:**
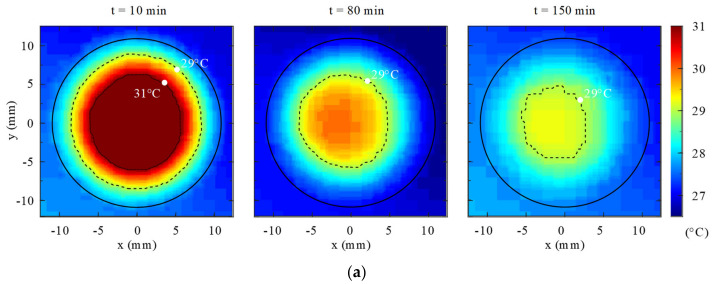

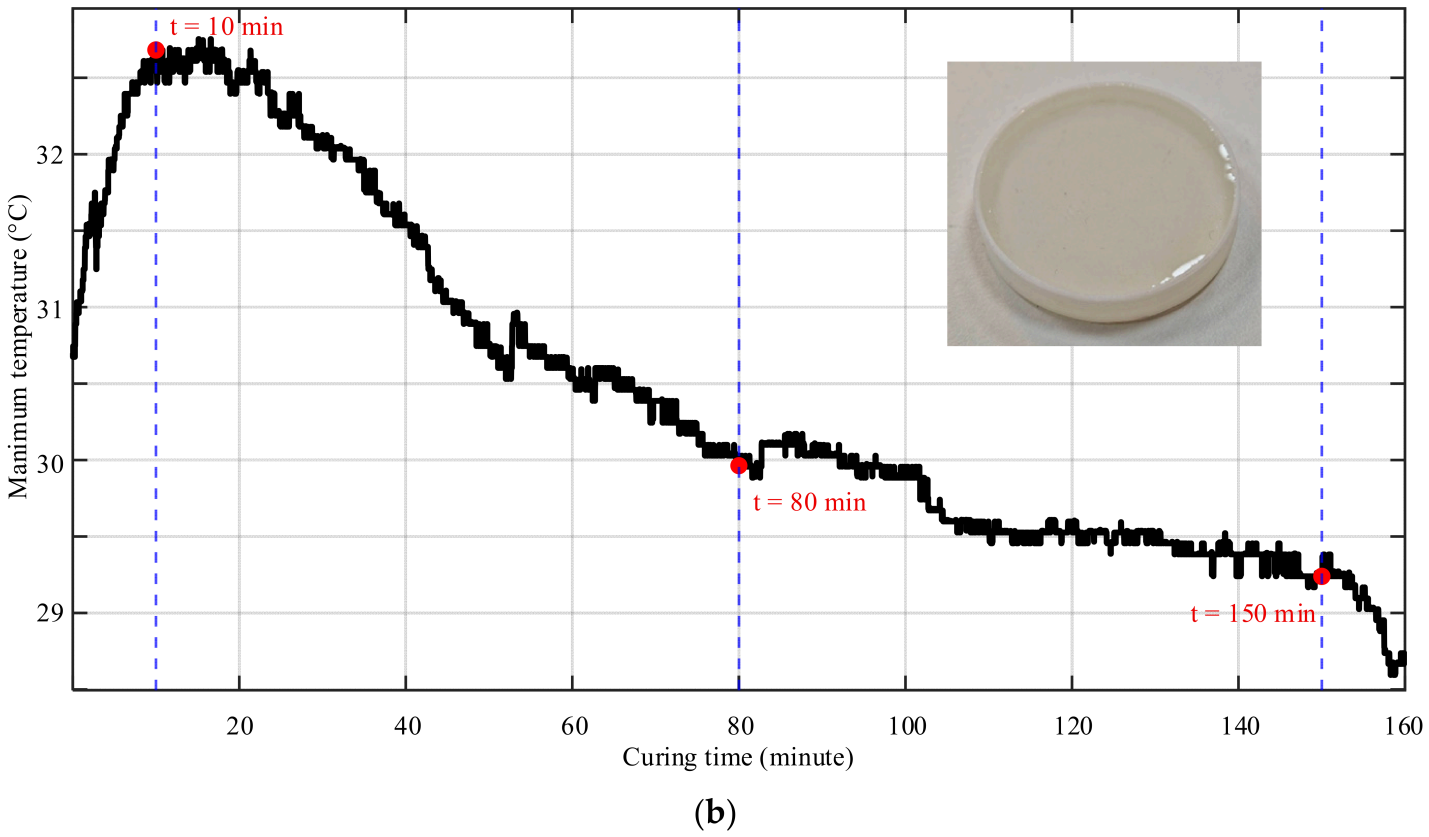
Thermal response of the two-component epoxy resin during confined curing. (**a**) Temperature evolution as a function of time, showing both the gradual heating phase and the subsequent thermal relaxation stage. (**b**) Maximum temperature recorded during the curing cycle. The results highlight the lower peak temperature and longer thermal timescale of epoxy curing relative to the UV-curable system.

In contrast to the UV-curable system, the epoxy resin exhibited a substantially longer curing timescale. As shown in Figure 7, the epoxy temperature increased to approximately 32.75 °C and then decreased gradually to about 28.5 °C over 160 min. The thermal profile is consistent with a partially cured state at approximately 80 min and an effectively completed cure by approximately 160 min under the tested conditions. By comparison, the UV-curable resin cooled from approximately 38 °C to 28 °C within about 2.5 min, indicating a much faster thermal recovery. This contrast highlights the distinct process trade-off between the two systems: the epoxy resin produced a lower thermal peak but required a much longer interruption to the fabrication sequence.

#### 4.3.3. Material Uniformity and Structural Continuity

Visual inspection of cured epoxy regions revealed a uniform, homogeneous appearance, with no evidence of layer-wise stratification. Bulk curing therefore promotes continuous material formation without discretized interfaces.

From an interfacial perspective, the reduced shrinkage rate and lower peak temperature are expected to reduce the likelihood of exceeding the stress and energy thresholds described in Equations (5)–(7), which is consistent with the observed resistance to delamination-like behavior under comparable confinement conditions.

#### 4.3.4. Implications for In Situ Casting Integration

Together, these results show that the epoxy system occupies a different operating regime from the UV-curable system within the present hybrid FDM workflow. Under the tested confined in situ casting conditions, the epoxy resin imposed a lower thermal peak on the surrounding FDM structure than the UV-curable system, but its substantially longer curing duration introduced a longer interruption to the print–pause–dispense–cure–resume sequence. The practical limitation of the epoxy system in the present workflow is therefore not thermal intensity but reduced temporal compatibility with layer-wise deposition.

This interpretation should be understood specifically within the context of the present experimental setup and process sequence. The results do not imply that chemically cured systems are broadly unsuitable for additive manufacturing, but rather that, under the tested in situ casting conditions, the lower thermal severity of the epoxy system is offset by a longer curing interruption. By contrast, the UV-curable system produced a shorter but thermally sharper event.

These findings suggest that material selection for in situ casting should consider not only final cured material properties, but also the compatibility of the curing mechanism with the intended fabrication cadence. In this sense, the present results highlight a process-level trade-off between thermal severity and interruption duration in hybrid FDM-integrated casting.

### 4.4. Layer-Wise Integration and Representative Structural Demonstration

**Figure 8:**
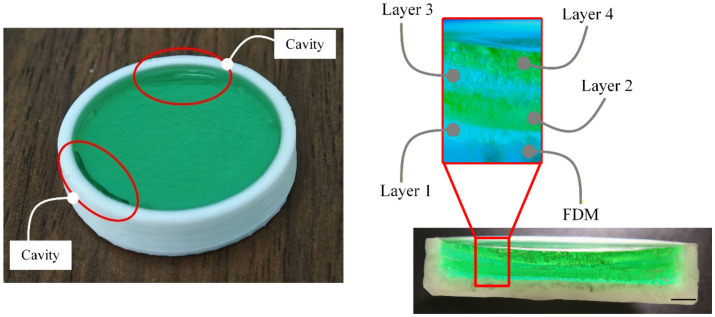
Layer-wise fabrication sequence incorporating dispensing-based in situ casting within an FDM printing workflow.

A layer-wise UV-based in situ casting strategy was evaluated within an FDM-fabricated confinement to determine whether repeated filling and curing steps could be integrated stably into the printing sequence. The fabrication sequence is summarized in Figure 8. A circular FDM-printed mold with a diameter of 30 mm, height of 5 mm, and wall thickness of 2 mm was used. As shown in Figure 8, the process consisted of repeated resin filling, staged UV curing, and thermal relaxation within the same printed cavity.

UV-curable resin was dispensed into the cavity in discrete 1 mL layers, and each layer was cured before the next was introduced. To accommodate limited curing depth and reduce heat build-up, a staged ramp–pulse curing schedule was applied using 0.5 s pulses of progressively increasing intensity separated by 5 s dwell intervals. Extended cooling periods were required between curing stages; in a non-ventilated enclosure, a dwell time of 200 s was needed, whereas forced ambient airflow reduced this interval to 20 s.

Using this sequence, four cast layers were successfully stacked within the printed mold. Alternating blue and green UV resins were used to distinguish adjacent layers and assess continuity. The final structure showed clear layer definition without visible leakage, shell deformation, or gross interfacial instability. These results show that UV-based in situ casting can be integrated layer-by-layer within an FDM workflow when exposure intensity, fill volume, and thermal relaxation are coordinated explicitly.

To assess structural relevance, a representative linkage component was also fabricated using the same hybrid workflow. The base linkage was first printed by FDM with local cavities at the joint region, after which brass bush inserts were placed and the surrounding cavities were filled with selected polymer materials. The investigated cases included rigid and semi-rigid reinforcement materials as well as more compliant filling materials, allowing comparison across a range of structural responses. This demonstrated that confined casting could be applied to an application-relevant geometry rather than only to simple test molds.

Mechanical testing under uniaxial loading indicated that local reinforcement influenced the observed structural response of the linkage in the demonstration cases. Stiffer cast materials increased resistance to deformation, whereas more compliant filled regions produced lower stiffness and greater flexibility. The demonstration cases suggest that the choice of casting material may influence the part-level structural response of the hybrid linkage. However, the present study was designed as a representative proof-of-feasibility demonstration and not as a comprehensive comparative mechanical characterization. These results suggest that dispensing-based in situ casting can provide functional structural modification in addition to demonstrating process feasibility.

## 5. Simplified Analytical Interpretation

To support interpretation of the observed process trends, a simplified analytical description is introduced for UV curing depth, thermal response, curing-induced stress, and cavity filling. The aim is not to provide a complete multiphysics model, but to summarize the main physical factors influencing process feasibility. The definitions and physical meanings of all variables employed in this analytical framework are provided in Table 2.

### 5.1. UV Attenuation and Curing Depth [39,40]

For UV-curable resins, light penetration decreases with depth according to an exponential attenuation law,

(1) $$\left(z\right)=I_{0}e^{-\alpha z}$$

where *I*_0_ denotes the incident UV intensity at the resin surface, *α* is the effective absorption coefficient, and *z* is the depth measured from the illuminated surface. Polymerization proceeds only when the accumulated local energy dose exceeds a critical threshold *D_crit_*, such that

(2) $$D\left(z\right)=\int_{0}^{t}I\left(z\right)dt\geq D_{crit}$$

which gives an approximate maximum curing depth

(3) $$z_{max}\approx \left(\frac{1}{\alpha }1/\alpha \right)ln\left(\frac{I_{0}t}{D_{crit}}\right)$$

This form is consistent with the observed diminishing increase in cured thickness at higher exposure levels.

### 5.2. Energy Balance and Peak Temperature [18,41,42]

A simplified transient energy balance for curing-induced heating may be written as

(4) $$\rho c_{p}\frac{\partial T}{\partial t}=k\nabla^{2}T+\Delta H\frac{\partial \phi }{\partial t}$$

where *ρ* is the resin density, *c*_*p*_ is the specific heat capacity, *k* is the thermal conductivity, Δ*H* is the heat of polymerization, and *ϕ* denotes the degree of cure. This relation reflects the balance between heat generation during curing and heat dissipation into the surroundings. It is consistent with the observed sensitivity of peak temperature to exposure intensity and the finite cooling interval measured after UV exposure.

### 5.3. Shrinkage-Induced Stress and Delamination Criterion [17,24,40,43]

Curing shrinkage may generate internal stress when the resin is constrained by the surrounding printed structure. A first-order estimate is

(5) $$\sigma_{S}\approx E\varepsilon_{s}$$

where *E* is the effective elastic modulus of the cured resin and *ε*_*s*_ is the volumetric shrinkage strain. Although the present study does not directly measure interfacial stress or fracture energy, this expression is useful for interpreting why thicker or more aggressively cured regions were more prone to visible warping and edge lifting.

### 5.4. Volumetric Filling Constraint [23,44]

For a cavity of volume *V*_*c*_, the dispensed volume *V*_*d*_ should satisfy

(6) $$V_{d}\approx V_{c}$$

to avoid underfilling or overflow. The volumetric flow rate may be written as

(7) $$Q=Av_{p}$$

where *A* is the effective syringe cross-sectional area and *v*_*p*_ is the plunger velocity. This constraint reflects the practical need to match dispensing conditions to cavity geometry and material behavior.

### 5.5. Practical Interpretation

Taken together, these simplified expressions provide a compact interpretation of the observed process behavior: UV curing is depth-limited by attenuation, thermal load depends on curing rate and dissipation, shrinkage contributes to deformation risk, and stable filling depends on matching dispensed volume to cavity capacity. These considerations are not intended as a predictive model for all cases, but as an analytical framework for interpreting the experimentally observed process limits.

## 6. Practical Process Guidelines

For UV-curable systems, cast volume per step should remain within the effective curing depth, and thick regions should be formed by layer-wise filling and curing rather than aggressive single-step exposure. Staged UV pulses with intermediate dwell times are preferable for limiting thermal accumulation and visible deformation. Active cooling can further reduce the required dwell interval.

For two-component systems, bulk filling is practical because optical penetration is not limiting. However, the longer curing time must be incorporated into the process schedule, so these systems are more suitable when longer pauses are acceptable.

Across both curing mechanisms, dispensed volume should match cavity capacity closely to avoid underfilling or overflow. Printed confinement walls must be robust enough to withstand curing-induced thermal and mechanical effects. Overall, stable implementation requires coordinated print–pause–dispense–cure–resume sequencing in which curing schedule, cooling interval, and fill volume are defined during process planning.

## 7. Discussion

The results show that dispensing-based in situ casting can be integrated into an FDM workflow, but the feasible operating window depends strongly on curing mechanism, thermal recovery, and cavity-level process control. The comparison between the UV-curable resin and the two-component epoxy makes this particularly clear. Although both systems could be introduced into printed confinement geometries, they imposed different constraints on process continuity and therefore are suited to different modes of hybrid fabrication.

For the UV-curable resin, the main limitation was the coupling between curing depth, peak temperature, and visible deformation. Increasing exposure increased cured thickness but also increased thermal loading and the likelihood of warping or edge lifting. This made aggressive single-step curing unsuitable for stable integration, particularly when repeated casting steps were required within the same printed structure. The thermal imaging results further showed that localized heating persisted beyond the exposure period, indicating that cooling time must be treated as part of the fabrication sequence rather than as a secondary consideration. In this sense, UV-based in situ casting is compatible with FDM when the process is discretized into small fill volumes, staged exposure, and explicit thermal relaxation intervals.

The two-component epoxy followed a different operating regime. Because curing was not limited by optical penetration, the resin could be introduced as a bulk-filled region under confinement. The associated thermal peak was lower than that of the UV-curable system, reducing the instantaneous thermal burden on the printed shell. However, this advantage was offset by a substantially longer curing time, which interrupted the layer-wise FDM sequence for a much longer period. The epoxy therefore shifts the process constraint from optical and thermal intensity toward temporal compatibility. This distinction is important because it shows that material selection for in situ casting cannot be based only on final cured properties; it must also account for how the curing mechanism fits within the timing requirements of the additive workflow.

A central outcome of the study is that curing-induced heat must be treated as a first-order constraint in hybrid fabrication. The thermal images showed radial heat dissipation from the cured region into the surrounding printed shell, confirming that the confinement geometry also functions as part of the thermal boundary condition. As a result, the cavity design, shell thickness, and cooling environment all influence how quickly the system can return to a state compatible with resumed printing. This was demonstrated directly in the layer-wise integration experiments, where forced airflow reduced the required dwell time substantially. The practical implication is that print continuation depends not only on whether curing occurs successfully, but also on whether the thermal state of the printed structure has sufficiently recovered.

Visible deformation was also shown to be a useful practical indicator of process instability. In the present study, deformation was evaluated through experimentally observable outcomes such as warping, edge lifting, and loss of flatness rather than through direct interfacial mechanics measurement. This is an important limitation, but it does not reduce the process relevance of the observations. In a hybrid printing workflow, such deformation may lead directly to leakage, dimensional loss, or poor compatibility with subsequent deposited layers. Accordingly, visible deformation can serve as a meaningful engineering criterion when screening feasible operating conditions, especially in early-stage process development.

The layer-wise integration experiment showed that repeated in situ casting can be performed within a single FDM-printed cavity when curing depth, fill volume, and thermal relaxation are coordinated explicitly. The successful stacking of four UV-cured layers demonstrated that the process is not limited to isolated cavity filling but can be extended to sequential build-up of cast regions during fabrication. This result is significant because it moves the method from a single-event filling operation toward a truly integrated hybrid workflow. At the same time, the need for staged curing and dwell intervals suggests that this capability comes at the cost of added process complexity and longer fabrication time.

The representative linkage demonstration showed that the approach also has structural relevance beyond process feasibility. By combining printed thermoplastic geometry, local polymer filling, and brass inserts, the workflow enabled changes in part-level stiffness and deformation response. The investigated cases showed that rigid and semi-rigid filling materials increased resistance to deformation, whereas more compliant fillings preserved greater flexibility. Although this was not intended as a full mechanical qualification study, it confirms that local casting can be used to tailor the structural response of an FDM-fabricated component. This makes the process especially relevant to applications where extrusion-only FDM is insufficient to provide the required local density, reinforcement, or insert retention.

Several limitations of the present work should be acknowledged. First, the study was limited to one representative UV-curable resin and one representative two-component epoxy system. Broader generalization will require validation across additional polymer systems with different viscosities, curing kinetics, shrinkage behavior, and thermal properties [45,46]. Second, the fundamental experiments were performed using simple confinement geometries to isolate key process variables. While this was useful for interpretation, more complex cavities may introduce additional flow, thermal, and stability effects. Third, the present study emphasized experimentally observable process behavior rather than full rheological, interfacial, or fracture-mechanics characterization. Fourth, the dispensing platform was used as a practical experimental tool and was not benchmarked against alternative metering architectures. Finally, the mechanical demonstration was limited to a representative linkage component and did not include fatigue, impact, environmental durability, or throughput benchmarking relative to conventional fabrication routes.

Overall, the results indicate that dispensing-based in situ casting is a viable hybrid operation within FDM when curing-dependent thermal and temporal constraints are treated explicitly in process planning. The UV-curable system favored rapid localized solidification but required staged exposure and cooling to maintain stability. The two-component epoxy enabled bulk filling with lower peak temperature but imposed a longer process interruption. The practical value of the method therefore lies not in replacing FDM as a general-purpose fabrication route, but in enabling localized process capabilities that are difficult to obtain using extrusion-only deposition.

## 8. Conclusions

This study investigated dispensing-based in situ casting integrated within an FDM workflow, with emphasis on curing-dependent constraints relevant to hybrid polymer fabrication. The results show that UV-curable resin can be incorporated successfully when curing is discretized and thermal relaxation is included between steps, whereas two-component epoxy enables bulk filling at lower peak temperature but requires much longer curing time. Infrared thermal imaging demonstrated that localized heating and finite cooling time are important constraints for print continuation. A staged layer-wise UV strategy enabled successful multi-layer casting within an FDM-printed confinement, and a representative linkage demonstration showed that localized casting can alter structural response through selective reinforcement.

These results indicate that dispensing-based in situ casting is a practical hybrid operation when thermal, temporal, and filling constraints are treated explicitly in process design. Its value lies in enabling localized capabilities that are difficult to achieve using extrusion-only FDM. Future work should extend the method to broader polymer systems, more complex cavity geometries, closed-loop thermal control, and clearer comparison of process time and part-level performance relative to conventional fabrication routes.

## Figures and Tables

**Table 1:** Representative comparison of additive, subtractive, and formative manufacturing routes relevant to hybrid polymer fabrication.

Manufacturing Process	Additive		Subtractive (CNC)	Formative
	FDM	SLA		
Material	Thermoplastics (ABS, PLA, PETG)	Photopolymer resins	Metals, plastics, wood	Thermosets (epoxy, polyurethane), metals
Process	Layer-by-layer extrusion	Layer-by-layer curing	Material removal from solid stock	Casting into molds
Pre-Process and Post-Process	Heating: △ Sand: ✗ Clean: ✗ Active cure: ✗ Coating: △	Heating: △ Sand: △ Clean: ✓ Active cure: ✓ Coating: △	Heating: ✗ Sand: ✓ Clean: ✓ Active cure: ✗ Coating: △	Heating: ✓ Sand: ✗ Clean: ✗ Active cure: △ Coating: △
Digital Fabrication (Manual Intervention)	✓	✓	✓	✓
Tensile Strength (MPa)	44 (ABS)	50 (Standard resin)	310 (Al 6061-T6)	60–75 (epoxy)
Elastic Modulus (GPa)	2.1 (ABS)	2.8–3.2 (Standard resin)	68.9 (Al 6061-T6)	2.5–3.5 (epoxy)
Surface roughness Ra (µm)	10–25 (across layers) 1–3 (along layers)	1–5	<1	1–10
Density (g/cm^3^)	~1.04 (ABS)	~1.10–1.15 (Standard resin)	~2.70 (Al 6061-T6)	~1.10–1.30 (epoxy)

✓ Routinely required. △ Optional/case-dependent. ✗ Not typical. Representative values are provided for context: ABS (~44 MPa), epoxy (~65 MPa), composite stiffness (~2.5–3.0 GPa), surface roughness ranging from Ra ≈ 10–25 µm (FDM) to <1 µm (machined), and density variations arising from multi-material integration (ABS, epoxy, brass).

**Table 2:** Definitions of variables.

Symbol	Descriptions	Unit
*I(z)*	UV light intensity at depth *z*	W·m^−2^
*I*_0_	Incident UV intensity at the resin surface	W·m^−2^
*α*	Effective optical absorption coefficient of the resin	m^−1^
*z*	Depth from the illuminated surface	m
*t*	UV exposure time	s
*D(z)*	UV energy dose at depth *z*	J·m^−2^
*D*_crit_	Critical energy dose required for polymerization	J·m^−2^
*z*_max_	Maximum achievable curing depth	m
*ρ*	Density of the resin	kg·m^−3^
*c*_p_	Specific heat capacity of the resin	J·kg^−1^·K^−1^
*k*	Thermal conductivity of the resin	W·m^−1^·K^−1^
*T*	Temperature	K or °C
Δ*H*	Exothermic heat of polymerization	J·kg^−1^
*φ*	Degree of cure	–
*ε*_s_	Polymerization-induced volumetric shrinkage strain	–
*σ*_s_	Shrinkage-induced stress	Pa
*E*	Effective elastic modulus of the cured resin	Pa
*σ*_adh_	Interfacial adhesion strength	Pa
*G*	Energy release rate at the resin–substrate interface	J·m^−2^
*G*_c_	Critical energy release rate for interfacial delamination	J·m^−2^
*Q*	Volumetric flow rate during dispensing	m^3^·s^−1^
*A*	Effective cross-sectional area of syringe plunger	m^2^
*v*_p_	Syringe plunger velocity	m·s^−1^
*V*_d_	Dispensed resin volume	m^3^
*V*_c_	Target cavity volume	m^3^
*T*_crit_	Maximum allowable temperature for FDM compatibility	°C
*W*	Feasible process window for in situ casting	–

## Data Availability

The raw data supporting the conclusions of this article will be made available by the authors on request. (“https://youtu.be/Do7YRbkYg8k?si=8aaU_mTUvbLE_p9a (accessed on 26 February 2026)”).
